# Transportation of therapeutic and controlled drugs across international borders: a descriptive analysis of information available to travellers

**DOI:** 10.1093/inthealth/ihac014

**Published:** 2022-03-25

**Authors:** Jack R Kissane, Gerard T Flaherty

**Affiliations:** School of Pharmacology and Therapeutics, National University of Ireland Galway, Galway, Ireland; School of Medicine, National University of Ireland Galway, Galway, Ireland

**Keywords:** controlled drugs, international travel, narcotics, psychotropic agents, regulations, therapeutic drugs

## Abstract

**Background:**

Transportation of medications across borders can present challenges for international travellers. We investigated the provision of official country-specific information available to individuals travelling with medications.

**Methods:**

Data from official government sources relating to restrictions on the transportation of medications for personal use were analysed to determine compliance with international recommendations and identify variation in practice between countries.

**Results:**

Schedule 1 drug classifications and import restrictions for narcotic and psychotropic drugs varied widely between countries. Some countries do not conform to recommendations from the International Narcotics Control Board.

**Conclusions:**

Healthcare professionals should be broadly aware of country-specific restrictions on therapeutic drug transportation and be able to direct travellers to definitive sources of information.

## Introduction

The International Narcotics Control Board (INCB) is an independent treaty body that operates worldwide. The laws that determine the drugs and drug quantities that may be imported into most countries are based on a list of recommendations set out by the INCB.^1^ The extent to which countries across the world comply with these recommendations varies significantly.^2^ International travellers would benefit from greater accessibility and uniformity in the rules and regulations that govern the medications they may lawfully transport into a country, particularly if their trip involves visits to multiple destinations. Violation of border customs laws may result in severe penalties, including spot fines and imprisonment, as well as travel delays, separation from other members of a travelling party and significant personal embarrassment and psychological stress. This pilot study aimed to collate and critically analyse official country-specific medication transportation customs data derived from publicly available sources.

## Methods

Data relating to the transportation of medications across borders were extracted from publicly available sources for the top five countries for inbound international tourism in 2019 within each continent (North and South America were combined), according to the 2020 United Nations World Tourism Organisation World Tourism Barometer and Statistical Annex. The standard INCB template (https://www.incb.org/documents/Travellers/files/NCA_ENG_2016.pdf) was used to record official government information for individual countries. Supplementary information was also extracted where this was available. Google Translate (https://translate.google.com/) was used to translate relevant information to the English language as appropriate.

## Results

Table 1 presents details of the information extracted from the top five most visited countries in each region. Most countries (64%, n=16) had an official government website containing relevant information on the subject of medication transportation across their borders. Five of these websites were not available in the English language although partial translations were achieved using Google Translate. The majority of the countries examined (76%, n=19) used the standard INCB template to communicate their import restrictions for narcotic and psychotropic drugs. Six of those 19 countries (32%) listed their prohibited substances for importation on the template itself. Thailand was the only country that listed all their prohibited substances on the template. Thirty-six percent (n=9) of the countries adhered to the INCB-recommended 30-d maximum import supply for narcotic and psychotropic drugs. A valid medical prescription was required at border customs by 56% (n=14) of the countries. There was a requirement for a doctor's certificate to be endorsed by the traveller's national health authorities in 32% (n=8) of countries. A minority of countries (24%, n=6) mandated a certificate issued by the health authorities of the destination country. The majority of countries (68%, n=17) publicly listed their prohibited substances, which ranged from a total of 3 (China) to 1547 (Canada) agents.

**Table 1.** Current country-level recommendations on carriage of personal medications

Country	Standard INCB template in use	Adherence to INCB-recommended maximum import quantities^a^	Valid medical prescription required	Certificate endorsed by health authorities of country of residence	Certificate issued by health authorities of destination country	Presentation of original prescription at customs of destination country	Government website available	Information available in English	Number of prohibited substances listed
**Europe**									
France	Yes	Yes	Yes	No	No	No	Yes	No	207
Spain	Yes	Yes	No	Yes	No	No	No	N/A	N/A
Italy	Yes	Yes	Yes	Yes	No	No	No	N/A	N/A
Turkey	Yes	No	No	No	No	Yes	Yes	No	N/A
Germany	Yes	Yes	Yes	Yes	No	No	Yes	Yes	186
**Asia**									
China	No	N/A	N/A	N/A	N/A	N/A	No	N/A	3
Thailand	Yes	Yes	Yes	Yes^b^	Yes^b^	Yes^c^	Yes	No	118
Japan	Yes	No	No	Yes^c^	Yes^b^	No	Yes	Yes	7
Malaysia	Yes	Yes	Yes	No	No	Yes	Yes	Yes	7
Hong Kong	Yes	No	Yes	No	No	No	Yes	Yes	184
**Americas**									
USA	Yes	No	Yes	No	No	Yes	Yes	Yes	245
Mexico	Yes	No	Yes	No	No	Yes	Yes	No	22
Canada	Yes	Yes	No	No	No	No	Yes	Yes	1547
Argentina	Yes	Yes	Yes	No	No	No	Yes	No	2
Brazil	Yes	No	Yes	No	No	No	Yes	Yes	N/A
**Africa**									
Egypt	No	N/A	N/A	N/A	N/A	N/A	No	N/A	N/A
Morocco	Yes	No	Yes	Yes	Yes	No	No	N/A	N/A
South Africa	Yes	No	Yes	Yes	Yes	Yes	No	N/A	5
Tunisia	Yes	No	Yes	No	Yes	No	No	N/A	N/A
Algeria	Yes	No	Yes	Yes	Yes	Yes	No	N/A	N/A
**Oceania**									
Australia	No	N/A	N/A	N/A	N/A	N/A	Yes	Yes	130
New Zealand	Yes	Yes	No	No	No	No	Yes	Yes	253
Fiji	No	N/A	N/A	N/A	N/A	N/A	Yes	Yes	7
Papua New Guinea	No	N/A	N/A	N/A	N/A	N/A	Yes	Yes	5
Samoa	No	N/A	N/A	N/A	N/A	N/A	No	N/A	253^d^

Abbreviation: N/A, not applicable.

a 30-d supply for narcotics and psychotropics.

b Narcotics, not psychotropics.

c Psychotropics, not narcotics.

d Number of substances reportedly similar to New Zealand; the list is not updated regularly.

## Discussion

International travellers are typically advised to carry adequate quantities of medications for personal use in their original labelled containers and, in the case of psychotropics, narcotic analgesics or certain substances such as pseudoephedrine,^3^ to ensure that they comply with any restrictions in place at their destination country's borders. We investigated the extent to which a sample of popular travel destinations differed in terms of the restrictions they applied and whether the INCB recommendations were implemented in each case. We found considerable variation in the application of these recommendations with potential for confusion arising among travellers who may not be familiar with country-specific requirements. In most cases, travellers must carry a medical prescription for the drugs they wish to import. A previous study found that none of the countries assessed permitted the importation of narcotic or psychotropic drugs without documentation that signified personal ownership, irrespective of medication quantity.^4^ We found that some countries, including Australia, China and Fiji, do not issue their regulations to the INCB in the form of a template. Countries that follow the INCB 30-d quantities of psychotropics/narcotics recommendation were under-represented in our study. Goodyer and Rajani previously reported that of the 184 countries studied, only 56 had made submission to the INCB, with information for only a further 14 easily identified online.^2^

A variety of restrictions and quantities of narcotic or psychotropic drugs was identified that did not always comply with the recommendations of the INCB. The list of prohibited substances published by each country varied significantly. Some INCB recommendations on prohibited substances (e.g. cannabinoids) may be outdated as they rely on older legislation. Countries in Oceania had the least amount of INCB-related information publicly available. By contrast, African countries required the most stringent INCB-related documentation on arrival. This could be potentially hazardous for unwary international tourists. No official customs website was found for the five African countries assessed, and only South Africa had information available on prohibited substances for importation. It may be reasonable to expect a consensus on INCB-related documentation within the EU bloc. However, four of the five European countries assessed follow the INCB 30-d recommendation. No relevant customs website could be identified for either Spain or Italy despite these countries having high volumes of inbound tourism. Information on prohibited substances for importation to these countries was also not identified.

Canada was found to classify the highest number of schedule 1 drugs. There were six times as many prohibited substances listed by Canadian authorities than the next highest ranked country (New Zealand). Over-the-counter medications like ibuprofen are deemed to be schedule 1 drugs in Canada in their extra-strength formulations. Vaccines are listed as schedule 1 drugs, and this expands the scope of their list greatly. Countries like New Zealand and Thailand have a multi-tiered system for prohibited substances. New Zealand operates a class A, B, C system, where the possession of a drug in any class is unauthorised. Thailand operates prohibitive drug classes based on whether the drug is narcotic or psychotropic in nature.

The INCB should continue to advise countries to adopt their template strategy in an attempt to harmonise communication with the travelling public. The country-specific drug importation database of the International Society of Travel Medicine is a valuable resource compiled by its pharmacist professional group but it will require ongoing investment to maintain its currency.^5^

Our study has several limitations. We selected countries based on the annual volume of inbound international tourism within their continental regions. Many notable tourism destinations with restrictive importation laws, such as the United Arab Emirates, were excluded on this basis. While every attempt was made to ensure the currency of the data analysed, it is possible that individual countries may have recently updated the information they provide to international travellers. Our pilot study may stimulate completion of a larger scale analysis of country-level requirements for international travellers carrying medications for personal use.

## Conclusions

This study identified considerable differences in practice between individual countries in relation to the provision of information on medication transportation by international visitors. Greater adherence to the standardised approach favoured by the INCB would facilitate communication of this information to prospective travellers and to their travel health advisers. We recommend the adoption of a centralised repository of such information that would be updated at regular intervals.

## Data Availability

The data underlying this article are available and were derived primarily from sources in the public domain, including official government customs websites with the *.gov* suffix. Website addresses for individual country-level websites are available upon request from the corresponding author.
